# Measurement of social inequalities in health: concepts and methodological approaches in the Brazilian context^*^

**DOI:** 10.5123/S1679-49742018000100017

**Published:** 2018-04-05

**Authors:** Inácio Crochemore Mohnsam da Silva, Maria Clara Restrepo-Mendez, Janaína Calu Costa, Fernanda Ewerling, Franciele Hellwig, Leonardo Zanini Ferreira, Luis Paulo Vidaletti Ruas, Gary Joseph, Aluísio J.D. Barros

**Affiliations:** 1 Universidade Federal de Pelotas, Programa de Pós-graduação em Epidemiologia, Centro Internacional de Equidade em Saúde, Pelotas, RS, Brasil

**Keywords:** Health Inequalities/methods, Socioeconomic Factors, Measurements, Methods and Theories

## Abstract

This study aims to describe methodological approaches to measure and monitor health inequalities and to illustrate their applicability. The measures most frequently used in the literature were reviewed. Data on coverage and quality of antenatal care in Brazil, from the Demographic and Maternal and Child Health Survey (PNDS-2006) and from the National Health Survey (PNS2013), were used to illustrate their applicability. Absolute and relative measures of inequalities were presented, highlighting their complementary character. Despite the progress achieved at national level in antenatal care, important inequalities were still identified between population subgroups, with no change in the magnitude of the differences throughout the studied period. Brazil has important social inequalities, which consequently lead to health inequalities. Their description and monitoring are highly relevant to support polices focused on those vulnerable population groups who have been left behind.

## Introduction

Global, regional and national estimates on health indicators are essential when it comes to the assessment and monitoring of a country, as well as to guide the allocation of resources in public health.^1,2^ However, such estimates often hide significant differences among population subgroups.^2-4^ In addition, promoting equality among these subgroups and prioritizing the improvement of indicators among vulnerable groups may be an efficient strategy to improve the national indicators.^4-7^ The Sustainable Development Goals (SDG) proposed by the United Nations in 2015, on the basis of the Millennium Development Goals (MDG), emphasize the importance of universal health care and the need to tackle inequality.^5^

Even though inequality and iniquity are often presented as synonyms, they are in fact two different concepts. Inequalities in health refer to any differences that may be observed among subgroups (in terms of socioeconomic status, education level, place of residence, sex, among others) within a population.^4,8^ On the other hand, iniquities are differences (or inequalities) that are considered unjust or unfair.^3,8^ In this sense, researches must first search for evidence of inequality among the population subgroups, so that public health may then focus on tackling iniquity.

it is of the utmost importance that the evidences of health inequalities are presented and taken into consideration for the basis of public policies.

There are several methodological approaches and strategies used in the literature to describe and analyze the distribution of health problems with focus on inequalities.^4^ The main objective is the identification of the most vulnerable groups (or less favored) that must be prioritized for public health interventions.^3,4^ The most commonly studied inequalities are those regarding sex and/or gender, race/ethnicity/skin color, socioeconomic status, income groups, education level, area of residence (urban or rural) and geographic region.^4^

In Brazil, specifically, there are significant historical social inequalities.^9,10^ This is reflected in the populations’ health, which makes social determinants of health key players in this context.^10,11^ For example, the evidence on the progress achieved on women’s and children’s health since the 1990s is overwhelming, but large inequalities based on place of residence, income bracket and geographic region still remain.^12-15^ In this sense, it is of the utmost importance that the evidences of health inequalities are presented and taken into consideration for the basis of public policies.^10^

Therefore, methodological issues for the analysis and description of health inequalities, as previously discussed in the international literature, should be prioritized in the country. This study aims to describe methodological approaches to measure and monitor social inequalities in health and to illustrate their applicability, using data on the coverage and quality of antenatal care in Brazil from the Demographic and Maternal and Child Health Survey (*Pesquisa Nacional de Demografia e Saúde da Criança e da Mulher*; PNDS-2006) and the National Health Survey (*Pesquisa Nacional de Saúde*; PNS-2013).

## Methodological approaches to measure health inequalities

We reviewed the methodological approaches to measure health inequalities most frequently found in the literature.^16,17^ Secondary data from two nationwide Brazilian surveys, the 2006 PNDS and the 2013 PNS, were used to illustrate the methodological approaches, to measure and understand health inequalities, as well as the evolution of the inequalities over time.

The 2006 PNDS is included in the 5^th^ phase of the Measure DHS (Demographic and Health Survey) project,^18^ which investigates the health and nutrition of women at reproductive age (15 to 49 years old) and children aged less than 5 years, in low and middle income countries.^19^ The PNS is a nationwide survey that focus on the health of Brazilian adults over 18, and has methodological characteristics similar to those of PNDS. Both surveys were conducted with multiple stages samplings and performed similarly, which favors their comparability.^20^ Information regarding women aged 18 to 49 who had a live birth in the last two years preceding the survey were included in the analysis. Among those women who had more than one child during the analysis period, only the information regarding the last pregnancy was included. Therefore, the analyzed sample was comprised of 1,440 women on PNDS, and 1,918 on PNS.

Information on antenatal care quality indicators found on both surveys were used to illustrate the methodological approaches. The indicators used were the six or more antenatal care visits during the pregnancy, as well as the quality of the antenatal aantenatalcare. The number of visits was categorized (<6 or ≥6) according to current recommendations from the Ministry of Health.^21^ The quality of the care was define as the performance of six procedures during the antenatal care visit as the minimum prescribed for an adequate antenatal care.^21^ This information was standardized and available on both surveys: (i) six or more antenatal care visits; (ii) first visit held in the first trimester of pregnancy; (iii) blood pressure measured in at least one visit; (iv) weight measured in at least one visit; (v) blood exams; and (vi) urine exams. Only women who reported undergoing all these six procedures were considered as having an adequate antenatal care.

The proportion of women who had six or more antenatal care visits and who had access to an adequate antenatal care was calculated at national level, and in subgroups according to area of residence (urban or rural) and household wealth quintiles.

The household wealth quintiles were calculated based on specific information of each survey regarding personal goods and characteristics of the household, using principal component analysis (PCA). On the PNDS (2006), the variables used were: the system and supply of water and electricity; the number of rooms; the existence and type of bathroom in the household; the household building materials (roof, floors and walls); having a domestic worker; ownership (and quantity) of household appliances (radio, television, VCR/DVD player, telephone, fridge, freezer, vacuum cleaner, washing machine); internet access; and car ownership.

In the PNS (2013), the information used referred, again, to: the household building materials; having a domestic worker; and car and household appliances ownership (television, DVD player, cellphone, computer, fridge, microwave oven and washing machine). In each survey, the score obtained from the PCA for each household were divided into quintiles, among which quintile 1 (Q1) represents 20% of the households that have the worst socioeconomic status and are at the lowest limit of wealth distribution, whilst quintile 5 (Q5) represents 20% of the households with the best socioeconomic status. Women who had children in the two years preceding the survey were categorized according to the classification of their households and presented, to simplify, as the poorest 20% (Q1) and the wealthiest 20% (Q5). The variables *area of residence and wealth quintiles* are called stratification variables, as they allow us to divide the sampling into groups (or strata), to assess and compare the results between subgroups.

The analytical procedures that identify and describe inequalities between groups are described below, along with the results.

## Applications: measurement and interpretation of health inequalities

Table 1 shows the proportion of women who had at least six antenatal care visits and those who presented adequate quality of antenatal care. The table also shows estimates stratified according to area of residence, wealth quintile, as well as absolute and relative measures of inequality. In 2006 and 2013, 76.9% (95%CI 72.8; 81.0) and 81.6% (95%CI 78.8; 84.5) of interviewed women had at least six antenatal care visits each, as recommended by the Ministry of Health. An increase was observed in the quality of antenatal care visits: in 2006, 60.6% (95%CI 56.6; 64.6) of pregnant women had adequate quality antenatal care, whilst in 2013 this percentage reached 73.9% (95%CI 70.6; 77.3).

**Table 1:** National coverage of six or more antenatal care visits and of adequate quality of antenatal care in Brazil, stratified according to area of residence and household wealth, and their respective absolute and relative measures of inequalities, Brazil, 2006 and 2013

Six or more antenatal care visits							
Strata	2006				2013		
	N	%	95%CI^a^	N	%	95%CI^a^
**National**	1,440	76.9	72.8; 81.0	1,918	81.6	78.8; 84.5
**Rural**	263	67.5	61.7; 73.4	303	73.8	65.7; 81.9
**Urban**	1,178	79.0	74.2; 83.8	1,615	83.1	80.1; 86.1
**Urban/rural difference**		11.5	3.8; 19.2	-	9.3	0.5; 18.1
**Urban/rural ratio**	411	1.2	1.0; 1.3	-	1.1	1.0; 1.3
**Q1 (20% poorest)**	378	67.3	61.7; 72.8	525	66.1	59.4; 72.7
**Q2**	263	72.3	63.4; 81.2	372	81.9	75.4; 88.3
**Q3**	239	79.1	69.5; 88.8	339	86.9	81.8; 91.9
**Q4**	150	90.8	83.6; 98.1	351	89.6	84.6; 94.6
**Q5 (20% wealthiest)**	-	88.7	78.2; 99.1	331	92.2	88.3; 96.0
**Difference Q5-Q1 (p.p.)^b^**	-	21.4	8.3; 34.5	-	26.1	18.3; 33.9
**Ratio Q5/Q1**	^-^	1.3	1.1; 1.5	-	1.4	1.2; 1.6
**CIX^c^**	-	7.1	4.7; 9.4	-	7.0	4.9; 9.2
**SII^d^ (p.p.)^b^**	^-^	31.2	19.9; 42.5	-	34.2	24.3; 44.1
**Adequate quality of antenatal care**							
**Strata**	**2006**				**2013**		
	**N**	**(%)**	**95%CI^a^**	**N**	**(%)**	**95%CI^a^**
**National**	1,440	60.6	56.6; 64.6	1,851	73.9	70.6; 77.3
**Rural**	263	56.4	50.9; 61.9	293	66.2	57.8; 74.6
**Urban**	1,178	61.6	56.8; 66.3	1,558	75.4	71.8; 79.0
**Urban/rural difference**	411	5.2	-2.2; 12.6	-	9.2	0.0; 18.5
**Urban/rural ratio**	378	1.1	1.0; 1.2	-	1.1	1.0; 1.3
**Q1 (20% poorest)**	263	51.3	45.2; 57.3	496	60.3	53.5; 67.1
**Q2**	239	56.1	46.7; 9.3	358	74.4	67.5; 81.3
**Q3**	150	62.9	53.3; 9.6	332	77.7	70.8; 84.6
**Q4**	-	70.9	62.2; 8.7	345	79.6	72.4; 86.8
**Q5 (20% wealthiest)**	-	77.3	66.8; 10.5	322	84.5	77.2; 91.9
**Difference Q5-Q1 (p.p.)^b^**	-	26.0	12.7; 13.3	-	24.2	14.1; 34.3
**Ratio Q5/Q1**	-	1.5	1.2; 0.3	-	1.4	1.2; 1.6
**CIX^c^**	-	9.1	6.0; 12.1	-	6.8	4.1; 9.5
**SII^d^ (p.p.)^b^**		29.6	18.8; 40.4	-	29.5	18.1; 41.0

a 95%CI: confidence interval of 95%.

b p.p.: percentage points.

c CIX: Concentration Index.

d SII: Slope Index of Inequality.

However, it should be once more stressed that national estimates may hide important inequalities between subgroups, by describing the current situation as well as its evolutions over time. These inequalities can be expresses in both relative and absolute measures,^17^ as described below.

### Measures of absolute inequalities

Absolute inequality is calculated as the difference between the measures of occurrence (prevalence, incidence, mortality) between groups, that is, by subtracting the extreme values. Results are presented in percentage points (p.p.) or following the same multiplier factor – for example, by 1,000, by 10,000, etc. Two widely used examples are the prevalence differences between the 20% wealthiest (Q5) and the 20% poorest (Q1), or the subtraction of indicator estimates in urban areas by the estimates of the same indicator in rural areas.

The Slope Index of Inequality (SII) is another measure for absolute inequality, used specifically for ordinal variables of stratification (usually socioeconomic the entire distribution of the stratification variable using indicators such as income groups, wealth and literacy the adequate regression model.^4,16,17^ Therefore, the SII rates). It represents absolute difference, in predicted is calculated as the difference, in percentage points, values, of a health indicator between the most privileged between the estimated values for the extreme groups individuals and the less privileged individuals in terms of a given stratification variable. Although the SII was of socioeconomic indicators, taking into consideration conceived based in a linear regression, in general the logistic regression is more adequate for its calculation because usually it is applied to coverage of indicators and prevalence of health outcomes, avoiding linear predictions out of the boundaries of an expected interval of a proportion (from 0 to 100).^16^

With regard to the proportions, both the absolute differences between group and the SII vary from -100 to 100 p.p., and values close to zero are expected when there is no inequality. Positive values reveal that the health indicator, be it the coverage of an intervention or the prevalence of a health risk, is more frequent in the most privileged group – for example, in the wealthiest group or the group with higher education. This is considered a “pro-rich” inequality. Negative values show that the health indicator is more prevalent in the less privileged group – for example, in the poorest group or the group with lower education, constituting “pro-poor” inequalities. Further examples of the differences and interpretations of coverage analysis and health outcomes can be found in Barros and Victora.^16^

In the data analyzed for this study, the absolute difference estimates among the groups found that the coverage of at least six antenatal care visits among women living in urban areas was, in 2013, of 9.3 p.p. (95%CI 0.5; 18.1) higher than among those living in rural areas. The inequality was even higher when the extreme wealth quintiles (Q5-Q1) were taken into consideration, showing that women in wealthiest families had a prevalence 26.1 p.p. (95%CI 18.3; 33.9) higher than those of poorest families. Similar results were found when analyzing the quality of antenatal care in 2013 (Table 1).

**Figure 1:**
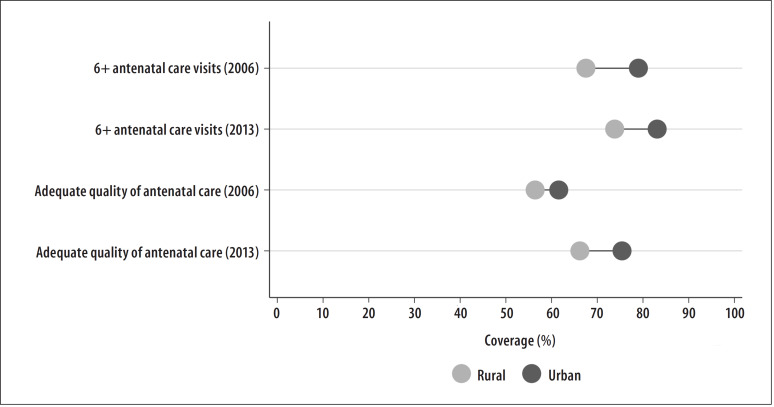
– Coverage of at least six antenatal care visits and adequate quality of antenatal care, according to area of residence, Brazil, 2006 and 2013

Note: The dots show the average coverage in each category (urban/rural). The horizontal lines connect the average coverage in urban areas (light gray) to the rural areas (dark gray). The distance between circles represents the absolute inequality. The bigger the line between the groups, the higher the absolute inequality.

**Figure 2:**
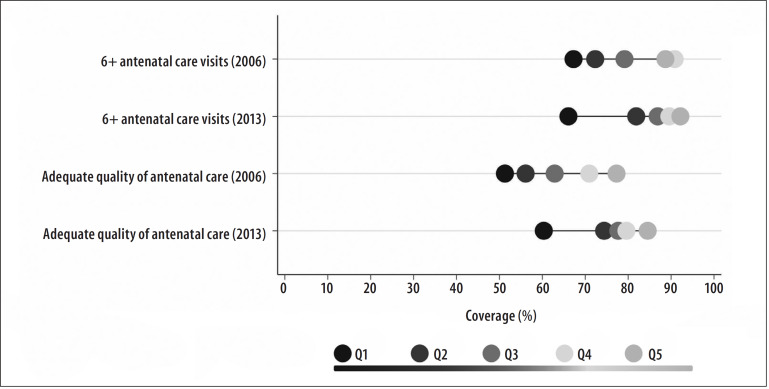
– Coverage of at least six antenatal care visits and adequate quality of antenatal care, according to household wealth quintiles, Brazil, 2006 and 2013

Note: The dots show the average coverage in each wealth quintile. Q1 represents the poorest 20% and Q5 represents the wealthiest 20%. The distance between quintiles 1 and 5 represents absolute inequality.

**Figure 3:**
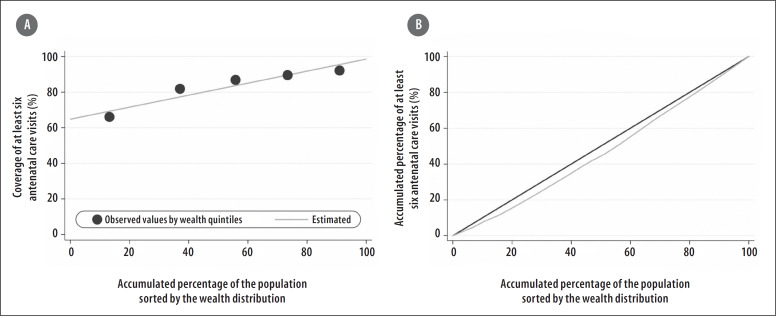
– (A) Slope Index of Inequality (SII), of at least six antenatal care visits, Brazil, 2013; (B) Concentration Index (CIX), of at least six antenatal care visits, Brazil, 2013

These differences between subgroups may also be clearly and simply illustrated using Equiplot graphs (www.equidade.org/equiplot), shown in Figures 1 and 2. Regarding the socioeconomic differences evidenced in the wealth quintiles analysis, the SII results found deeper inequalities, precisely because they considered all the data distribution, not only the extreme groups, as exemplified in Figure 3(A). In this way, the absolute difference in the prevalence of at least six antenatal care visits among women whose families belong to the wealthiest quintile and those of women who belong to the poorest quintile was of 34.2 p.p. in 2013, according to Table 1 and Figure 3(A).

Note:

In figure 3(A), the dots show the average coverage observed in each wealth quintile and the line represents the estimated values. The difference between the extreme values of this distribution indicates the value of the SII (34.2 p.p.).

In Figure 3(B), the CIX corresponds to twice times the area formed between the curve, which indicates the observed coverage, and the straight diagonal line, which represents perfect equality (CIX = 7.0).

The absolute measures of inequality have great interpretability potential. The “gap” between rich and poor, or in how much the coverage of a given subgroup needs to be increased so that equality may be reached, is an extremely important information and easily comprehensible to public health managers.

## Measures of relative inequalities

The ratio between the estimates of two extremes of the stratification variables is the simplest relative inequality measure. It can be calculated by, for example, dividing the values (prevalence, coverage etc.) that correspond to the wealthiest group (Q5) by the value of the poorest group (Q1). This gives the excess percentage of category when compared with another or how higher the prevalence in a group is in comparison with the other.

Another measure of relative inequality is the Concentration Index (CIX), which, like the SII, takes into consideration all the categories of the stratification variable. The CIX is similar to the Gini Coefficient – it varies from -1 to +1, assumes zero as equality, and, the further the values are from zero, the highest the relative inequality is.^16,17,22^ The value of CIX corresponds to twice times the area between a diagonal line that represents perfect equality among the groups and the curve that expresses the coverage observed for each cumulative percentage of the population studied (Figure 3(B). When coverage is greater among the wealthiest, the area generated is under the diagonal line, and when coverage is higher among the poorest, the opposite is true. When coverage is measured in relation to wealth, just as in the absolute measure, positive values indicate “pro-rich” differences and negative values mean “propoor” differences.^16^ Some studies also present the CIX as values multiplied by 100, to improve data visualization, along with the measures of absolute inequality, none of which alters interpretation.

When analyzing national data on the women who had at least six antenatal care visits in 2013, relative differences regarding the number of visits and the areas of residence were not observed (urban/rural ratio: 1.1; 95%CI 1.0; 1.3). However, in the CIX analysis, evidence of “pro-rich” inequalities were identified (CIX: 7.0; 95%CI 4.9; 9.2), as shown in Figure 3(B) and Table 1.

The relative measures can highlight how unequal the estimates among groups are, even being very sensitive to the occurrence of the outcome studied. Indicators with lower frequencies may present high relative inequalities, overestimating these differences in a public health overview. Similarly, high prevalence outcomes may present important inequalities, but are not grasped by relative measures. For example, an intervention that has a coverage of 5% among the richest and 1% among the poorest group will show an absolute inequality of 4 p.p. and a relative inequality that is 5-fold (i.e., 5%/1%), or 400% higher towards the rich (i.e., [5-1] x 100%). Meanwhile, in another scenario, where the intervention has a coverage of 50% among the richest and 10% among the poorest, the same 400% relative inequality is found, but with an absolute inequality that is 40 p.p. higher among the richest. Both situations have the same relative inequality, but represent two very dissimilar situations in terms of implications for public health. This does not mean that absolute measures are more adequate than relative ones, or vice-versa. Actually, absolute and relative measures are complementary should both be used together.

It is worth mentioning that, for the interpretation of absolute and relative inequalities, it is necessary to make a distinction between differences expressed in percentages (%) and in percentage points (p.p.). In 2013, the percentage of women who reported having at least six antenatal care visits was 92.2% among the richest (Q5) and 66.1% among the poorest ones (Q1), revealing a difference of 26.1 p.p. (absolute difference). The ratio between these two values produces a result equals to 1.4 (95%CI 1.2: 1.6), revealing a prevalence that is approximately 40% higher among the wealthiest women (relative difference).

## Patterns of inequality

Once the absolute or relative inequalities have been identified, the patterns that these inequalities present may also be studied. This might help with the planning of more efficient approaches to improve coverage and reduce inequalities.^16^ Two patterns of inequality should be highlighted as they are very common in in low and middle income countries are: bottom and top inequalities. The bottom inequality pattern is identified when the coverage of a given intervention includes most of the population, but cannot reach the less privileged group, such as the lowest socioeconomic quintile. In real terms, this pattern shows a markedly lower coverage for the poorest quintile in comparison with the others. On the other hand, in the top inequality pattern the opposite phenomenon occurs, where a given intervention that such cover the entire population ends up contemplating mostly those with higher economic levels.^16,22,23^ Both patterns show clearly in the data analysis for antenatal care in Brazil (Figure 2). In 2006, both adequate quality of antenatal care, and, especially, coverage of six or more antenatal care visits, presented top inequality pattern, with markedly higher coverage among the two wealthiest quintiles (the richest 40%). In 2013, the inequality pattern was inverted, becoming a bottom inequality pattern, where only the poorest women were left markedly behind (Figure 2). This type of transition over time is unfortunately expected. In general, the wealthiest/better-educated groups are the first to achieve access to interventions that will, throughout time, become available to the poorest groups.^24^

## Trends in inequalities

The evaluation of inequalities trends is necessary for assessing whether historical differences among population subgroups are changing over time. The main methodological approach consists of evaluating the time trends of summary indexes of inequalities (such as the SII and the CIX), which reinforces once again how complementary the absolute and relative measures are to each other.^16,25^ In a hypothetical scenario in which the coverage of a given intervention is, at the beginning of the evaluation, 40% among the richest and of 20% among the poorest, a relative measure based on the ratio between both groups would identify a prevalence twice as high among the richest, whilst an absolute measure of inequality would find that the richest have a coverage that is 20 p.p. higher than the poorest. If the coverage increased to 60% among the richest and 30% among the poorest, by evaluating solely the evolution of relative inequality, the inequality would be identified as stable over time (the coverage would still be two-fold among the richest). However, the absolute measure could identify an important increase in the inequality, from 20 to 30 p.p.

Another possibility to evaluate the evolution of inequalities is the study of time trends of the indicators stratified by population subgroups. That way, the inequalities may be identified both by the difference in the magnitude of estimates changes over time between groups (for example, an increase in the coverage of all groups, but with a higher magnitude among the poorest in comparison with the richest), as well as by the existence of changes among a single population group (for example, the coverage increases only among the poorest group).^26^ In both examples, a decrease in inequality would be observed. Using this approach, Victora et al. (2017) assessed a time trend between 1993 and 2014 of a composite indicator of health coverage for women and children (composite coverage index – CCI), stratified into two groups based on wealth. The authors showed that the difference in coverage between the rich and the poor decreased substantially only in middle income countries, when compared to low income countries. In middle income countries, the magnitude of the increase in coverage of that composite indicator was higher among the poor, when compared to the rich.^27^

In the analysis of antenatal care in Brazil presented in this article, there are no significant differences, in terms of relative and absolute inequalities, in the evaluated indicators, between the years of 2006 and 2013 (Table 1).

## Discussion

The methodological approaches to measure and monitor inequalities applied to the analysis of coverage of at least six antenatal care visits and adequate quality of antenatal care, as recommended by the Ministry of Health, demonstrated inequalities on both years studied (2006 and 2013). Moreover, any decrease in the magnitude of these differences was observed in the period. However, the discussion in this article will focus on the methodological approaches presented, which comprise the main possibilities for the identification and description of health inequalities. Absolute and relative measures were described and exemplified, as well as inequality patterns and their monitoring over time.

The analysis of inequalities of the health care of women and children can be based on single indicators, as the coverage of a given health intervention (skilled attendant at birth, number of antenatal care visits), or on composite indicators, as the quality of antenatal care. The indicator of quality of antenatal care, in this context, gathered only the information found in the surveys available in Brazil, and did not try to contemplate all the criteria involved in the evaluation of antenatal care quality. It should be noted that, regardless of its limitations, this indicator enable us to demonstrated important differences regarding socioeconomic conditions and area of residence in Brazil.

Composite indicators are usually robust in the identifications of health inequalities, since the coverage of some specific indicators may present greater variability among population subgroups, while putting these indicators together may support the identification of patterns. Among composite indicators of women’s and children’s health, one of the most frequently used is the CCI, which was developed by the Countdown initiative in 2015.^28^ This composite indicator contemplates ongoing reproductive, maternal, neonatal and child health care, evaluating family planning, skilled attendant at birth, antenatal visits, vaccines (DPT3, BCG and measles), and care for diarrhea and suspected cases of pneumonia.^28,^ ^29^

Findings from inequalities monitoring point toward the need to evaluate their various dimensions. Although the original analysis in this study was focused only on a socioeconomic indicator and on the area of residence, differences between geographic areas, ethnicities, age groups and sex/gender categories should be studied. Besides these stratifications, commonly found in the literature, others may be used according to the specificities of the studied population. The levels of women’s empowerment in a given region, for example, can be used to evaluate the differences in the access to different services and interventions by women with different level of independence and decision power.^30^

When it comes to socioeconomic indicators, there is a wide range of options that have been used for studying inequalities.^30-34^ Information on personal property and consumption have been considered good options to measure socioeconomic levels, since household and individual income are difficult to measure in a populationbased survey. In this context, socioeconomic indicators based in household goods have been a good alternative, due to the relative feasibility in measuring and comparing the information between different populations in the study.^32,33^ Finally, another socioeconomic indicator usually collected in population-based surveys that can be used in inequalities analysis is the education level. In low and middle income countries, like Brazil, the higher the education level of a population is, the higher their socioeconomic level will be. However, socioeconomic and education levels may not present precisely the same results in some health outcomes, especially those most influenced by knowledge level and information access.

Another relevant aspect in evaluating inequalities is the data availability, as well as comparability between different studies. For women’s and children’s health, there are several standardized surveys that have been conducted periodically in low and middle income countries. The Demographic Health Surveys (DHS) (available in: http://dhsprogram.com) and the Multiple Indicator Cluster Surveys (MICS) (available in: http://mics.unicef.org) are the main source of data on the subject in these countries. In Brazil, two DHS type surveys were conducted (1986 and 1996). Although the most recent national health surveys (e.g., PNDS and PNS) meet the countries’ specific demands, they do not cover all the topics on women’s and children’s health that are internationally relevant, and do not provide the necessary standardization for several indictors. Therefore, comparative analysis with other countries’ studies, or even researches for the evaluation of time trends in Brazil, become impossible. In this study, for example, aiming to present and discuss the main methodological approaches to measure health inequalities, we used two surveys, which were not designed with the same objectives, and as such presented differences in sampling procedures and target population.

## Conclusion

In this study, different approaches to identify and present health inequalities were shown, without attempting to end all possibilities of analysis. It should be noted that, when describing and monitoring talhealth inequalities, the complementarity of absolute and relative measures must be considered for a comprehensive description of inequalities. When choosing an approach, several factors must be taken into consideration, guided by the research question to answer or by the immediate answer to public authorities it seeks to provide.

Summary measures of inequality, such as SII and CIX, which consider all the distribution of data, and not only assess extreme groups, can be measures methodologically more appropriate. On the other hand, measures based on single ratios or differences of population groups may seem overly simplified, but have a great advantage when disclosing the results for specific audiences, as they are easily understood. Despite there has been an increase in the production of academic and scientific researches on health inequalities, dialoguing with health managers remains a challenge, and, thus, simpler measures are important instruments to favor these relations. Finally, the main objective of the monitoring of health inequalities is to offer support to policies that aim to decrease inequality, and as such it is of utmost importance that the results are presented adequately and data interpreted strictly, according to the methodological approaches employed.
